# LncRNA HOTAIR regulates glucose transporter Glut1 expression and glucose uptake in macrophages during inflammation

**DOI:** 10.1038/s41598-020-80291-4

**Published:** 2021-01-08

**Authors:** Monira Obaid, S. M. Nashir Udden, Prasanna Alluri, Subhrangsu S. Mandal

**Affiliations:** 1 Department of Chemistry and Biochemistry, The University of Texas at Arlington, Arlington, TX 76019 USA; 2 Department of Radiation Oncology, The University of Texas Southwestern Medical Center, Dallas, TX 75390 USA

**Keywords:** Biochemistry, Immunology, Molecular biology

## Abstract

Inflammation plays central roles in the immune response. Inflammatory response normally requires higher energy and therefore is associated with glucose metabolism. Our recent study demonstrates that lncRNA HOTAIR plays key roles in NF-kB activation, cytokine expression, and inflammation. Here, we investigated if HOTAIR plays any role in the regulation of glucose metabolism in immune cells during inflammation. Our results demonstrate that LPS-induced inflammation induces the expression of glucose transporter isoform 1 (Glut1) which controls the glucose uptake in macrophages. LPS-induced Glut1 expression is regulated via NF-kB activation. Importantly, siRNA-mediated knockdown of HOTAIR suppressed the LPS-induced expression of Glut1 suggesting key roles of HOTAIR in LPS-induced Glut1 expression in macrophage. HOTAIR induces NF-kB activation, which in turn increases Glut1 expression in response to LPS. We also found that HOTAIR regulates glucose uptake in macrophages during LPS-induced inflammation and its knockdown decreases LPS-induced increased glucose uptake. HOTAIR also regulates other upstream regulators of glucose metabolism such as PTEN and HIF1α, suggesting its multimodal functions in glucose metabolism. Overall, our study demonstrated that lncRNA HOTAIR plays key roles in LPS-induced Glut1 expression and glucose uptake by activating NF-kB and hence HOTAIR regulates metabolic programming in immune cells potentially to meet the energy needs during the immune response.

## Introduction

Inflammation is a biological response of the immune system^1–3^. Inflammatory response is triggered by a variety of factors such as pathogenic infection and exposure to toxic compounds^3–6^. Activation of immune response removes injurious and toxic stimuli, helps healing, and thus vital to health^4,7–9^. However, uncontrolled inflammation may result in severe inflammatory diseases^10^. Macrophages play key roles in provide a first line of defense against pathogens, eliminate the foreign substances and apoptotic cells through phagocytosis, and keep tissues healthy^11,12^. Pattern recognition factors such as toll-like receptors (TLRs) and others expressed in the macrophage surfaces detect the danger signals in the surrounding environment, activate macrophages, and initiate the signaling cascade. There two major type of macrophage activation process, M1 and M2 types. For example, when macrophages detect inflammatory cytokines such as interferon-gamma (IFNγ) and tumor necrosis factor alpha (TNFα) or foreign material such as lipopolysaccharide (LPS), they become activated and undergo a phenotypic change resulting in activated M1 types macrophage polarization^12–14^. However, when they encounter anti-inflammatory cytokines such as IL-4 and IL-13, macrophages are polarized to M2 (suppressive) state^4,15^. M1 type activated macrophages secrete proinflammatory inflammatory cytokines and chemokines such as IL-1β, IL-6, TNFα, CXCL1, and others, and induce inflammatory immune responses^4,7^. However, M2 macrophages secret anti-inflammatory cytokines such as IL-10, IL-18, and suppress inflammation and facilitate tissue repair^4,15^. Interestingly, M1 macrophages preferentially metabolize glucose as an energy substrate, while M2 macrophages primarily utilize fatty acids to fuel cellular functions^16,17^. Therefore, glucose metabolism is central to the function of classically activated M1 macrophages and could be a potential target for modifying inflammatory responses^4,15–17^. Studies suggest that proinflammatory immune response in M1 macrophages enhances expression of glucose transporter isoform 1 (Glut1), glucose-6-phosphate dehydrogenase, hexokinase, and increases glucose uptake^16,18^ and elevated Glut1-driven glucose metabolism drives inflammatory immune responses in macrophage^19,20^.

Signaling mechanism associated with inflammation and immune response is very complex, follows different pathways and is widely associated with various factors^2,21–24^. These include activation of transcription factors such as NF-κB, activator protein 1 (AP1), interferon regulatory factors (IRFs), STAT 1/2 (signal transducer and activator of transcription) and many others^18,25,26^. These activated transcription factors, in turn, stimulate the expression of proinflammatory cytokines, chemokines, interferons and other pro-inflammatory mediators, and thus propagating cellular inflammation^27–29^. Emerging evidences suggest that, in addition to proteins, noncoding RNAs (ncRNA) play critical roles in variety of cell signaling process including in regulation of gene expression and immune response^30–32^. NcRNAs are recently discovered class of transcripts that coded by the genome, but mostly remain untranslated into proteins^33–35^. Even though, they are untranslated, ncRNAs are functional and integral components of signaling processes^36–38^. Recent studies indicate that ncRNAs play important roles in regulating aerobic glycolysis in cancer cells^39–41^. For instance, miR-199a, miR-138, miR-150 and miR-532-5p inhibit the expression of Glut1^42–44^, while miR-130b, miR-19a, miR-19b and miR-301a induce Glut1 expression in epithelial cancer cells^31,45^. Recently, we discovered that a long noncoding RNA (lncRNA), HOTAIR, is involved in regulation of inflammatory immune responses in macrophages^30^. HOTAIR is an antisense lncRNA (2.2 kb long) which is well known for its function in gene silencing^46,47^. HOTAIR navigates polycomb repressive complex 2 (PRC2)mand lysine specific demethylase 1 (LSD1) complexes to their target genes promoters^48–50^. PRC2 and LSD1 recruitment introduce promoter histone H3K27-methylation and H3K4-demethylaytion, respectively and induce gene silencing^33,47^. HOTAIR is suppresses the tumor suppressor gene expression and it is overexpressed in variety of cancers^50,51^. Studies from our laboratory show that HOTAIR expression is elevated in breast cancer cells., its gene expression is regulated by estradiol^52,53^ and dysregulated by estrogenic endocrine disrupting chemicals (EDCs, e.g. BPA and DES)^52,54^. HOTAIR is an oncogenic lncRNA^51,55,56^. In addition to its well-worn functions in gene expression, HOTAIR is implicated in protein degradation associated with neuronal function and diseases^57–59^.

Our recent study demonstrated that HOTAIR plays critical roles in regulation of NF-κB activation via degradation of IκBα and regulate expression of cytokines and pro-inflammatory genes regulating inflammation and immune response^30^. Importantly, activation of immune cells in response to infection or other stressors is a metabolically expensive event^31,60,61^, and immune cells preferentially meet their energy needs by metabolizing glucose^62,63^. As HOTAIR plays critical role in NF-κB activation and inflammatory response in macrophage, we hypothesized that HOTAIR plays critical roles in inflammation induced metabolic reprogramming. Here, we explored any function of HOTAIR in glucose metabolism in macrophage during inflammation. Our study demonstrated that HOTAIR plays key roles in regulation of the expression of glucose transporter and glucose uptake, and hence regulates glucose metabolism in macrophages during LPS-induced inflammation and immune response.

## Materials and methods

### Mouse macrophage cell culture

RAW264.7 cells (mouse macrophages) were procured from American Type Cell Culture Collection. These cells were cultured in Dulbecco's modified Eagle's medium (DMEM; Sigma-Aldrich, St. Louis, MO) supplemented with 10% heat-inactivated FBS (Fetal bovine serum), 2 mM l-glutamine, 100 units/mL penicillin and 0.1 mg/mL streptomycin in a humidified incubator with 5% CO2 and 95% air at 37 °C^64^. Cells were counted to seed 2 × 10^6^ cells in 60 mm cell culture plates. After overnight incubation, macrophages were ready for treatment^30,65^.

### Cell treatment with lipopolysaccharide (LPS)

Macrophage cells were treated with ultrapure *E. coli*-derived LPS (Invivogen), 1.0 μg/mL, for different time periods. The concentration of LPS has been broadly reported to be used by other laboratories to induce immune and pro-inflammatory response in macrophages^30,65^. Cells were harvested for the preparation of RNA and protein analysis.

### RNA extraction and cDNA synthesis

Total RNA was extracted from the cells using TRIzol (Invitrogen) according to the manufacturer’s instructions^65^. Briefly RAW264.7 macrophages were harvested with TRIzol, mixed with chloroform and centrifuged at 12,000 rpm for 15 min. The aqueous layer was mixed with equal volume of 100% ethanol and centrifuged at 12,000 rpm for 10 min. After washing the pellet with 70% ethanol, purified RNA was dissolved in 30–50 μL of RNase-free water (Sigma) and quantified using a Nanodrop spectrophotometer. 1 μg of the isolated RNA was reverse transcribed into cDNA using iScript RT-supermix (Bio-Rad)^30,65^.

### Real time PCR

Real-time PCR was done using iTaq Universal SYBR Green Supermix (Bio-Rad), with gene specific PCR primers as listed in Table 1. The CFX96 real-time detection system (Bio-Rad) was used for RT-qPCR. Each experiment was repeated three times with three parallel replicates each time. Expression data were normalized to GAPDH and expressed as 2^−ΔCt^^66,67^.

**Table 1:** Sequences of primers.

Primers	Forward (5′–3′)	Reverse (5′–3′)
**PCR primers**		
HOTAIR	TCCAGATGGAAGGAACTCCAGACA	ATAGATGTGCGTGGTCAGATCGCT
IL-6	CAAGAAAGACAAAGCCAGAGTC	GAAATTGGGGTAGGAAGGAC
iNOS	TGTGACACACAGCGCTACAACA	GAAACTATGGAGCACAGCCACAT
Glut1	GCTGTGCTTATGGGCTTCTC	CACATACATGGGCACAAAGC
Glut2	CCCTGGGTACTCTTCACCAA	GCCAAGTAGGATGTGCCAAT
Glut3	CTGGGGTCACAGGTTAAGGA	ACAGAAGCCGCTCTCAGAAG
Glut4	ACTCTTGCCACACAGGCTCT	CCTTGCCCTGTCAGGTATGT
Glut5	TGTACCCCACCTCTCACTCC	CTCGGGTAGCAATGGACAGT
Pten	TGAGTTCCCTCAGCCATTGCCT	GAGGTTTCCTCTGGTCCTGGTA
HIF1α	CCTGCACTGAATCAAGAGGTTGC	CCATCAGAAGGACTTGCTGGCT
GAPDH	ACCCAGAAGACTGTGGATGG	CACATTGGGGGTAGGAACAC
**ChIP PCR primer**		
Glut1 promoter^a^	GCACACTTTCCCCTTCCTAGTT	AGACTCATGGGAAAATCCCACATT
**Antisense oligonucleotides**		
HOTAIR Antisense	5′-C*C*T*T*C*C*T*T*C*C*G*C*T*C*T*T*A*C*T*C*T-3′	
Scramble Antisense	5′-C*C*T*C*T*T*C*T*G*T*C*T*C*T*T*C*C*C*G*C*T-3′	

^a^ChIP PCR primers are flanked around the NF-κB binding site.

*All phosphorothioate linkages instead of regular phosphodiester bonds.

### Western blot analyses

The treated macrophage cells were washed with ice-cold PBS and then lysed in cell lysis buffer comprising 50 mM Tris–HCl (pH 8.0), 150 mM NaCl, 5 mM EDTA, 1% Igepal CA-630, 0.5% Na-deoxycholate, 2 mM Na_3_VO_4_, and complete protease inhibitor cocktail and phosphatase inhibitor cocktail (Roche). The resulting cell lysates were centrifuged for 10 min at 13,000 rpm at 4 °C, and the protein concentrations in the supernatants were determined using a BCA protein assay kit (Pierce)^68^. 20 μg proteins were loaded onto 10% SDS-PAGE gels, followed by electro-transfer onto PVDF-membrane (Immobilon-P, Millipore). The membranes were blocked in 1 × TBST (0.1% Tween-20, 20 mM Tris–Cl (pH 8.0), and 150 mM NaCl) containing 3% skimmed milk and then incubated with the primary antibodies against IκBα (1:1000 dilution, 4814S, Cell Signaling), Glut 1 (1:1000 dilution, 12939S, Cell Signaling), Phospho-p65 (NF-κB subunit, 1:1000 dilution, 3033S, Cell Signaling), HIF-1α (1:1000 dilution, 14,179, Cell Signaling) and β -actin (1:1000 dilution, A2066, Sigma) overnight at 4 °C. Membranes were washed 3 times (1xTBST), incubated with horseradish peroxidase-conjugated secondary antibodies for 1 h at room temperature and then washed 3 times (1 × TBST). Lastly, immunoreactive proteins were detected using ECL -super signal west femto substrate reagent (Thermo Scientific)^30,68^. The amount has been quantified by Image Lab 5.2.1 software.

### Chromatin Immunoprecipitation (ChIP) assay

The ChIP assay was done as described earlier^30,54^. The cells were cross-linked with 1% formaldehyde for 10 min at 37 °C, washed twice with ice-cold PBS and harvested using SDS lysis buffer (1% SDS, 10 mM EDTA, 50 mM Tris. HCl, pH 8.1) supplemented with complete protease inhibitor (Roche). Cells were sonicated to shear the chromatin (∼ 200–300 bp range). The fragmented chromatin was pre-cleared with protein G agarose beads (16–266, EMD Millipore) and subjected to immunoprecipitation using antibodies specific to CBP (A22, Santa Cruz Biotechnology, Sc369), Phospho-p65 (3033, Cell Signaling), histone H3K4-trimethyl (07–473, EMD-Millipore), histone acetylation (06–599, EMD-Millipore), RNA Pol II (8WG16, Abcam), and β-actin (A2066, Sigma). Immunoprecipitated chromatin was washed, de-crosslinked and deproteinized at 65 °C in presence of 5 M NaCl followed by incubation with proteinase K (Sigma) at 45 °C for 1 h^30,54,69^. Purified ChIP DNA fragments were analyzed by semi-quantitative PCR and real-time PCR using primers spanning NF-κB binding sites present in the Glut1 promoter (Table 1).

### SiRNA-mediated knockdown of HOTAIR

For the siRNA transfection, RAW 264.7 cells were grown up to 60% confluency in 60 mm culture plates and transfected with HOTAIR-siRNA, a pool of 4 different siRNA constructs (SI05685183, SI05685190, SI05685197, and SI05685204 Qiagen)^57^ and scramble siRNA (1027310 Qiagen) independently using GenMute siRNA and DNA transfection reagent (SL100568, SignaGen Laboratories) according to the manufacturer’s protocol.A cocktail of transfection reagent and siRNA was made prior to transfection. Initially, 12 μL (12 μg) of GenMute reagent was mixed with 300 μL DMEM (minus FBS and antibiotics) in an eppendorf tube. SiRNA was mixed with 100 μL DMEM (without supplements) in a separate eppendorf. Then the diluted siRNA solution was mixed with diluted GenMute reagents and allowed to stand for 30 min in the dark. In the intervening time, cells were washed twice with supplement-free DMEM and then 1.7 mL of supplement-free DMEM was added to each cell culture plate. Finally, siRNA transfection reagents cocktail was applied to the cell plates, mixed gently and incubated for 48 h. Cells were then stimulated with LPS (1 μg/mL) for specified time period and then harvested for RNA/protein extraction or for ChIP assays^30,69^.

### Antisense-mediated knockdown of HOTAIR

For the antisense transfection, RAW 264.7 cells were grown up to 60% confluency in 60 mm culture plates and transfected with HOTAIR-antisense (HOTAIR-AS) and scramble antisense (Scr-AS, no homology to HOTAIR) oligonucleotides^56,57^ independently (Table 1) using GenMute siRNA and DNA transfection reagent (SL100568, SignaGen Laboratories) according to the manufacturer’s protocol. A cocktail of transfection reagent and antisense oligonucleotides was made prior to transfection. Initially, 12 μL (12 μg) of GenMute reagent was mixed with 300 μL DMEM (minus FBS and antibiotics) in an eppendorf tube. Antisense oligonucleotides were mixed with 100 μL DMEM (without supplements) in a separate eppendorf. Then the diluted antisense solution was mixed with diluted GenMute reagents and allowed to stand for 30 min in the dark. In the intervening time, cells were washed twice with supplement-free DMEM and then 1.7 mL of supplement-free DMEM was added to each cell culture plate. Finally, antisense transfection reagents cocktail was applied to the cell plates, mixed gently and incubated for 48 h. Cells were then stimulated with LPS (1 μg/mL) for specified time period and then harvested for RNA extraction.

### NF-κB inhibition assay

RAW264.7 macrophages (2 × 10^6^) were seeded in 60 mm cell culture plates. After overnight incubation cells were initially treated with IKKβ inhibitor (25 μM, SC-514, Sigma) for 1 h to inhibit NF-kB signaling pathway and then cells were treated with LPS (1 μg/mL) and incubated for additional period of time 4 h^70^. Cells were harvested, total RNA was isolated using TRIzol reagent, reverse transcribed to cDNA and analyzed by qPCR. Protein was also extracted (SC-514 1 h and additional 1 h LPS treatment) after cell harvesting for Western blot^30^.

### Immunofluorescence microscopy analysis

For immunofluorescence staining of macrophages, cells were seeded on cover slips and fixed in 4% paraformaldehyde (PFA) for 15 min at room temperature, washed with 1X PBS (3 times for 5 min each) and blocked with 1X PBS containing 5% goat normal serum and 0.3% Triton-X100 for 1 h. The cells were then incubated with primary antibody (rabbit anti- Glut 1, 1:200, 12939S, Cell Signaling) overnight at 4 °C. After that the cells were washed 3 times with PBS followed by incubation with anti-rabbit Alexa Fluor 564 (Invitrogen), conjugated secondary antibody for 1 h at room temperature. Finally the cells were washed 3 times with PBS and mounted with mounting media containing DAPI. Images were taken by fluorescence microscope (Nikon ECLIPSE TE2000-U) utilizing a 63 × oil objective lens and quantified by ImageJ software^30^.

### Glucose uptake assay

Glucose uptake into the macrophages was determined using Glucose Uptake Assay Kit (Colorimetric, ab136955, Abcam) as per the manufacturer’s Instructions^19,45^. RAW264.7 macrophages and/or BMDMs were treated with HOTAIR siRNA and/or HOTAIR-antisense for 48 h and then seeded in 96 well plate in serum free medium. After overnight incubation cells were washed and incubated in 2% Bovine serum albumin (BSA) for 40 min. Then cells were stimulated with insulin (+/−), followed by 2-deoxyglucose addition for 20 min. 2-DG-6-phosphate (2-DG6P) standard curve was prepared. In this assay, the 2-DG6P was oxidized to generate NADPH, which was measured at 412 nm in a microplate reader^19,45^.

### Isolation and culture of primary macrophage (bone marrow derived macrophage, BMDM)

BMDM was isolated from mice bones and cultured as described earlier^65,71^. Wild-type (C57BL6/J) mice were purchased from Jackson Laboratory. All mice are maintained in a specific pathogen free (SPF) facility at UT Southwestern Medical center. All studies were approved by the Institutional Animal Care and Use Committee (IACUC) and were conducted in accordance with the IACUC guidelines, the National Institutes of Health Guide for the Care and Use of Laboratory Animals and *the ARRIVE guidelines*. For the isolation of BMDMs, femur and tibia were collected from mouse legs. Using 25G needle bone marrows were flushed out with Iscove's Modified Dulbecco's Medium (IMDM), (12440061, Life technologies) and processed for single cell suspension by passing through 22G needle two times. The suspension was centrifuged at 1000 RPM for 5 min. The pellet was re-suspended with BMDM culture media (L-cell-conditioned IMDM medium supplemented with 15% L929 supernatant, 10% FBS, 1% nonessential amino acid, and 1% penicillin–streptomycin) followed by seeding in three 150 mm culture dishes and cultured for 6 days to differentiate into macrophages, while at day 3, 10 mL fresh BMDM culture media was added into each plate. After day 6, the culture plate was washed with ice cold PBS and using cell scrapper cells were gently scraped with ice cold PBS. The BMDM was centrifuged at 1000 rpm for 5 min and re-suspended into BMDM media. The BMDM was counted and seeded in 6-well (2.5 × 10^6^/well) cell culture plates. After overnight incubation the BMDM was treated with LPS or HOTAIR siRNA and processed for further experiments.

### Statistical analysis

Each experiment was done in two or three replicates, and then cells were pooled (and treated as one sample), subjected to RNA extraction, RT-PCR, and ChIP analysis, and each experiment was repeated at least three times (n = 3). The real-time PCR analysis of such samples were done in three replicate reactions and repeated in all three independent experiments (n = 3). Data are presented as means ± SD (as stated in the figure legends). Statistical significance was determined by unpaired Student’s t test (GraphPad Prism 6), and P ≤ 0.05 was considered statistically significant^30^.

## Results

### LPS-induces GLUT1 expression in macrophage

**Figure 1:**
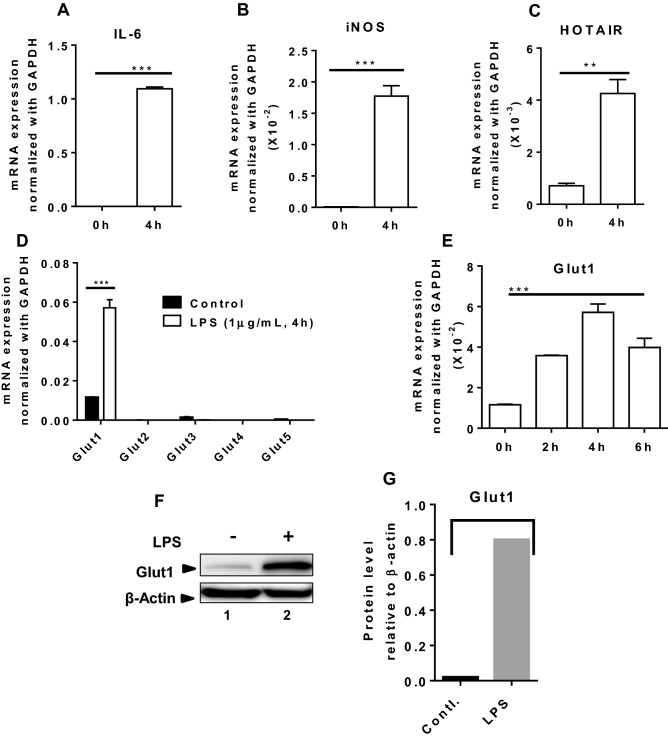
LPS induces Glut1 expression in macrophages. (**A**–**E**) RAW264.7 cells were treated with LPS (1 μg/mL) for 4 h (or different time periods), total RNA was isolated, reverse transcribed to cDNA and the expressions of IL-6, iNOS, HOTAIR, and different glucose transporters (Glut1, Glut2, Glut3, Glut4 and Glut5) were analyzed by RT-qPCR. GAPDH was used as control. The expression (relative to GAPDH) of IL-6, iNOS, HOTAIR, Glut1, Glut2, Glut3, Glut4 and Glut5 are shown in panels (**A**–**D**). The expression of Glut1 at different time periods of LPS-treatment is shown in panel (**E**). (**F**–**G**) LPS stimulated protein (1 h LPS-treatment) was extracted from macrophages and analyzed by Western blot using antibodies against Glut1 and β-actin (loading control). Quantifications (using ImageLab5.2.1software) is shown in panel (**G**). Each experiment was repeated at least with three parallel replicates. Data represent mean ± SD (n = 3); *p < 0.05, **p < 0.001, ***p < 0.0001.

Our recent studies demonstrate that lncRNA HOTAIR plays critical roles in inflammation and immune signaling and regulates cytokine expression via regulation of NF-κB activation^30^. As glucose metabolism is well-known to be elevated during inflammation^13,16,72^, we investigated if HOTAIR plays any roles in glucose uptake and metabolism. To investigate roles of HOTAIR in glucose metabolism during inflammation, initially, we examined if glucose transporters (Gluts) expression are affected upon lipopolysaccharide (LPS, present in membranes of gram negative bacteria) stimulation. We treated mouse macrophage cells (RAW264.7) with LPS and analyzed its impact on various Gluts expression. Briefly, RNA from the control and LPS-treated macrophages are reverse-transcribed into cDNA and analyzed by qPCR using primers specific to different glucose transporters (Glut1, Glut2, Glut3, Glut4, and Glut5). We also analyzed the expression of well-known inflammatory marker genes such as IL-6 and iNOS, and inflammatory noncoding RNA HOTAIR. As expected, the expression of IL-6, iNOS, and HOTAIR are significantly stimulated (by 9800, 272 and 6 folds, respectively) by LPS-treatment (Fig. 1A–C). Interestingly, expression of Glut1 is also significantly induced by LPS in macrophage, while the expression of other gluts (Glut2, Glut3, Glut4 and Glut5) were nominally affected (Fig. 1D). Time course analysis demonstrates that LPS-induced stimulation of Glut1 is maximum at 4 h (Fig. 1E). Western blot analysis also demonstrates that Glut1 expression is increased at the protein level upon treatment with LPS (Figs. 1F and S1, quantification in Fig. 1G). These observations demonstrate that Glut1, which is the primary glucose transporter expressed in macrophages, is upregulated upon LPS-induced inflammation and is potentially involved in glucose uptake and metabolism during inflammation. Notably, LPS-induced Glut1 expression has reported earlier and our observations are in agreement with the previous studies^13^.

### Glut1 expression is regulated by NF-κB during LPS stimulation

**Figure 2:**
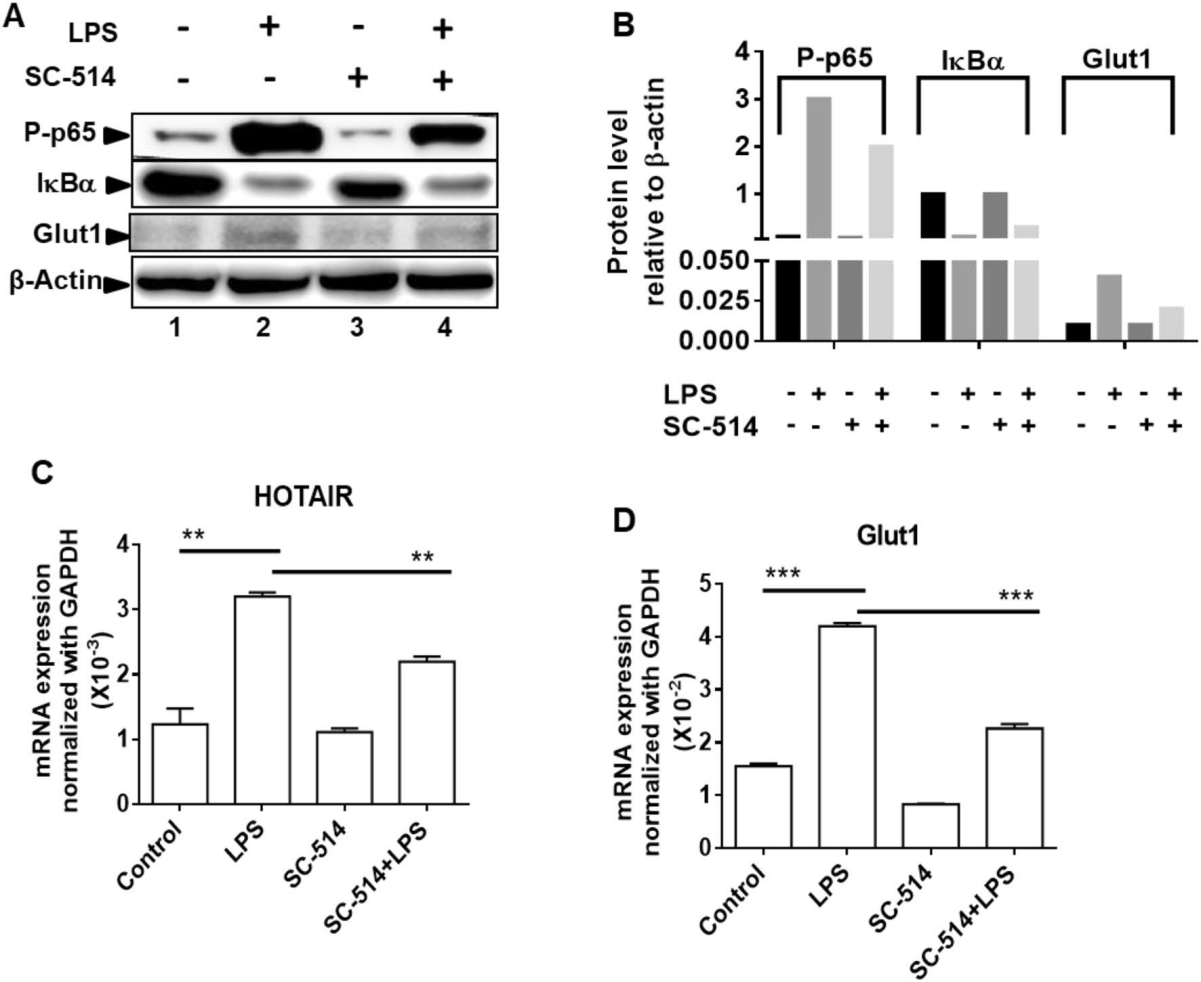
Inhibition of NF-κB downregulates LPS-induced Glut1 expression in macrophages. (**A**,**B**) RAW264.7 cells were initially treated with IKKβ-inhibitor SC-514 (for 1 h) and then treated with LPS for additional 1 h (for protein analysis) and 4 h (for RNA analysis). Proteins were analyzed by western blotting using antibodies against phospho-p65 (NF-κB subunit), IκBα, Glut1, and β-actin (loading control) (panel **A**, quantifications in panel **B**). (**C**,**D**) RNA was isolated from above treated and control cells. Expressions (relative to GAPDH) of HOTAIR, and Glut1 were measured by RT-qPCR. GAPDH was used as control. Data represent mean ± SD (n = 3); *p < 0.05, **p < 0.001, ***p < 0.0001.

The transcription factor NF-κB activation plays central roles in inflammation and immune response^7,27,73^. NF-κB activation is required for the expression of cytokine and pro-inflammatory genes^4,15^. Notably, under basal condition (in the absence of inflammation), NF-κB is complexed with IκBα and remains inactive^74^. However, upon inflammation, IκBα gets phosphorylated by the kinase IKKβ that results in its poly-ubiquitination followed by proteasomal degradation, and hence release of NF-κB (NF-κB activation). Therefore, inhibition of IKKβ would result in inhibition NF-κB activation^14,74,75^. To investigate if NF-κB activation is associated with LPS-induced Glut1 expression, we treated macrophages with an IKKβ kinase inhibitor (SC-514) and analyzed its impacts on LPS-induced Glut1 expression. Western blot analysis demonstrates that, upon treatment with LPS, the level of IκBα is decreased, while the level phospho-p65 (NF-κB subunit) is increased (compare lanes 1 and 2, Figs. 2A and S2, quantification in panel B). Notably, the LPS-induced IκBα degradation level as well as increased phospho-p65 levels, are reversed upon treatment with IKKβ-inhibitor (SC-514) (compare lanes 1, 2 and 4, Figs. 2A and S2, quantification in panel B). These observations suggest that LPS treatment resulted in NF-κB activation and this is inhibited by SC-514. Interestingly, RT-qPCR analysis demonstrates that LPS-treatment induced the expression of HOTAIR and upon IKKβ-inhibition (SC-514 treatment), the LPS-induced expression of HOTAIR is suppressed (Fig. 2C). Interestingly, the Glut1 expression is increased upon treatment with LPS and this LPS-induced Glut1 expression level is suppressed upon treatment with SC-514 (Fig. 2D). The LPS-induced increase in Glut1 protein level and its decrease upon SC-514-treatement is also evident in the Western blot (compare lanes 1, 2 and 4, Fig. 2A,B). These observations demonstrate that Glut1 expression is augmented upon LPS-induced inflammation and this is potentially regulated via NF-κB activation.

### HOTAIR regulates LPS-induced Glut1 expression

**Figure 3:**
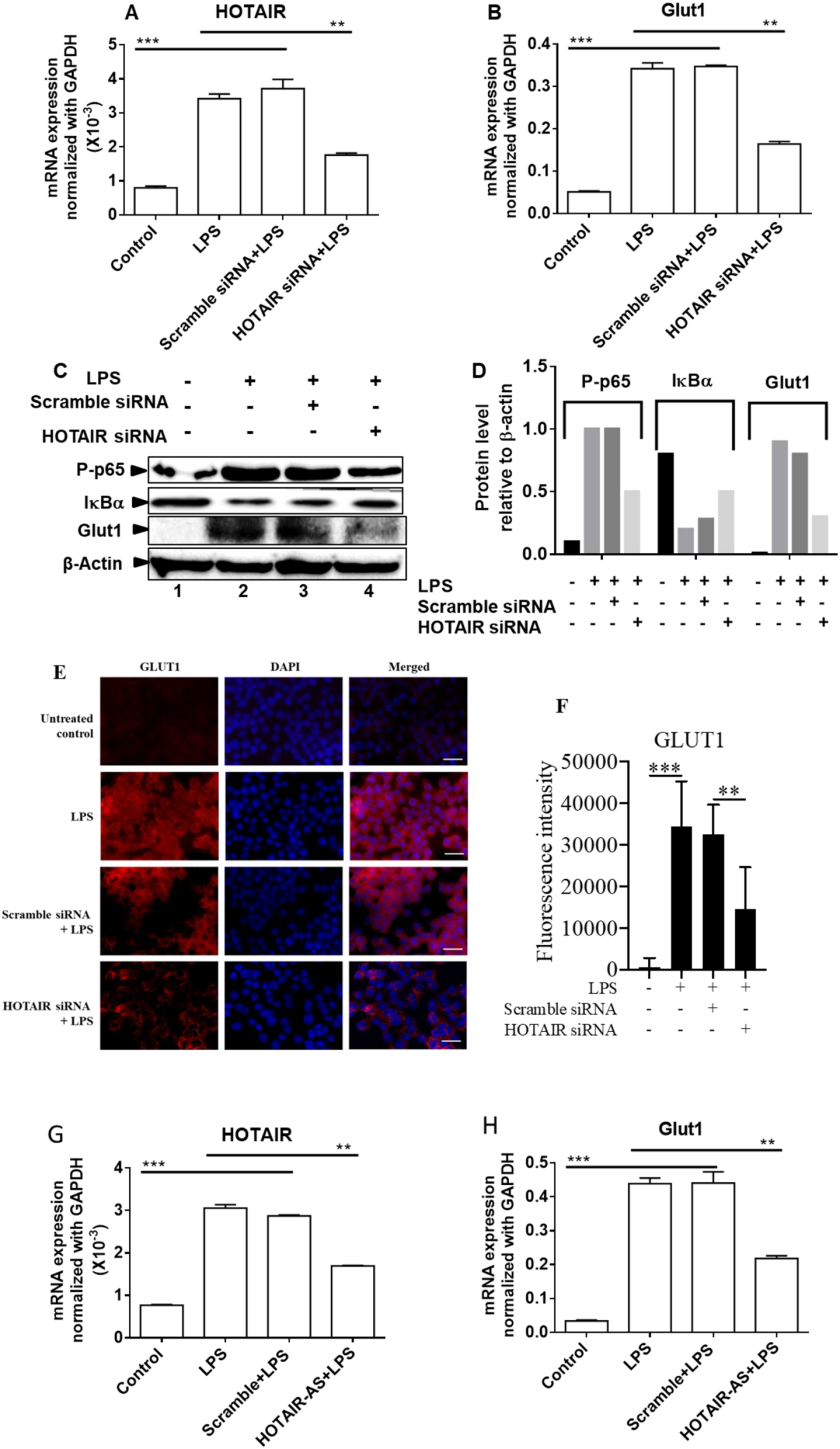
Knockdown of HOTAIR reduces LPS-induced Glut1 expression in macrophages. (**A**,**B**) RAW264.7 cells were transfected (48 h) with HOTAIR-siRNA and scramble siRNA followed by treatment with LPS. RNA was analyzed by RT-qPCR for the expression of HOTAIR and Glut1. GAPDH was used as control. Data represent mean ± SD (n = 3); *p < 0.05, **p < 0.001, ***p < 0.0001. (**C**,**D**) Proteins from HOTAIR-siRNA and scramble siRNA treatments (48 h) followed by 1 h LPS-treated RAW264.7 cells were analyzed by Western blotting using antibodies against phospho-p65 (NF-κB subunit), IκBα, Glut1, and β-actin (loading control) (panel **C**). The changes in amounts of NF-κB, IκBα, and Glut1 have been quantified by ImageLab5.2.1 software and shown in panel (**D**). (**E**,**F**) Immunofluorescene analysis: RAW 264.7 cells were transfected with HOTAIR siRNA and scramble siRNA (control) for 48 h and then treated with LPS (1 h), then fixed with paraformaldehyde and immunostained with antibody against Glut1, and counterstained with DAPI. Images were taken by fluorescence microscope (Nikon ECLIPSE TE2000-U) utilizing a 63 × oil objective lens (**E**) and fluorescence intensity showing the expression of Glut1 was quantified and plotted by ImageJ software (**F**). Bar is 100 uM. Data represent mean ± SD; *p < 0.05, **p < 0.001. (**G**,**H**). RAW264.7 cells were transfected (48 h) with HOTAIR- and scramble antisense oligonucleotide (separately) followed by treatment with LPS. RNA was analyzed by RT-qPCR for the expression of HOTAIR and Glut1. GAPDH was used as control. Data represent mean ± SD (n = 3); *p < 0.05, **p < 0.001, ***p < 0.0001.

Our recent studies demonstrated that HOTAIR plays key roles in NF-κB activation, cytokine expression, and inflammation^30^. Here to investigate if HOTAIR plays any roles in regulation of LPS-induced Glut1 expression, glucose uptake, and metabolism, we knocked down HOTAIR (using siRNA) in macrophages, then treated with LPS, and analyzed its impacts on Glut1 expression (RNA and protein levels). Briefly, RAW264.7 macrophages were transfected with HOTAIR- and scramble siRNAs (48 h) followed by treatment with LPS. RNA and proteins were isolated and analyzed by RT-qPCR and Western blot, respectively. Our RT-qPCR analysis demonstrated that the expression of HOTAIR is increased upon treatment with LPS and this LPS-induced expression of HOTAIR is significantly reduced (56%) upon application of HOTAIR siRNA (Fig. 3A). Scramble siRNA has no significant impact on HOTAIR expression level (Fig. 3A). Interestingly, RT-qPCR analysis also showed that Glut1 expression (mRNA level) is increased upon LPS-treatment and the LPS-induced Glut1 level was decreased upon HOTAIR-knockdown (Fig. 3B). Western blot analysis showed that Glut1 protein level is increased by LPS and that is decreased upon HOTAIR-knockdown (HOTAIR-siRNA treatment, Figs. 3C and S3, quantification in 3D). Western blot analysis also showed that LPS- treatment induced the degradation of IκBα and that is inhibited upon HOTAIR-knockdown (compare lanes 1, 2 and 4, Figs. 3C,D and S3). Concomitantly, the LPS-treatment also increased the phospho-p65 (NF-κB subunit) level and which is decreased under HOTAIR-knockdown conditions (compare lanes 1, 2 and 4, Figs. 3C,D and S3). Taken together, these observations demonstrate that LPS-induced Glut1 expression is regulated by HOTAIR in macrophages and this is mediated via NF-κB activation controlled by HOTAIR under LPS-stimulation.

To further understand the function of HOTAIR in Glut1 expression, we measured the expression of Glut1 in macrophages by using immunofluorescence assay. We knocked down HOTAIR in RAW264.7 cells by using HOTAIR-siRNA and scramble-siRNA (control) and then treated with LPS. Control and siRNA-treated cells were subjected to immunostaining with Glut1 antibody followed by Alexa Fluor 564 -conjugated secondary antibody and nuclear staining with DNA binding dye DAPI. Cells were then analyzed under a fluorescence microscope (Nikon ECLIPSE TE2000-U)^30^. We observed that Glut1 expression levels were low in the control cells (in the absence of LPS) and this was increased in upon stimulated with LPS (compare top two panels, Fig. 3E, quantification in panel F). Interestingly, upon HOTAIR-knockdown (HOTAIR-siRNA and LPS treatments), the level of LPS-induced Glut1 expression was decreased relative to LPS alone (Fig. 3E,F). Scramble siRNA has no significant impact on LPS-induced expression of Glut1 (Fig. 3E,F). These results further support our observation that HOTAIR is required for LPS-induced activation of Glut1 in macrophages.

To further address any off-target effects of HOTAIR-siRNA, we have also knocked down HOTAIR using a HOTAIR-specific antisense-oligonucleotide and analyzed its impacts on LPS-induced Glut1 expression. Briefly, RAW264.7 cells were transfected with HOTAIR-antisense and scramble-antisense (separately, as a negative control) for 48, then stimulated with LPS and RNA was analyzed by RT-qPCR. These analysis demonstrated that HOTAIR-antisense specially knocked down HOTAIR level and the knockdown of HOTAIR downregulated the LPS-induced expression of Glut1 (Fig. 3G–H). Our previous studies demonstrated that antisense-mediated knockdown of HOTAIR, down-regulates the LPS-induced increased phospho-NF-κB (phospho-p65) level via inhibition of LPS-induced I-κBα degradation^30^. Thus, using both siRNA and HOTAIR-antisense mediated knockdown experiments, we demonstrate that HOTAIR regulates Glut1 expression and this is likely via regulation of NF-κB activation. HOTAIR-mediated regulation of Glut1 expression suggests potential roles of HOTAIR in glucose uptake and metabolism under inflammation in immune cells.

### HOTAIR regulates LPS induced NF-κB recruitment in the Glut1 promoter

**Figure 4:**
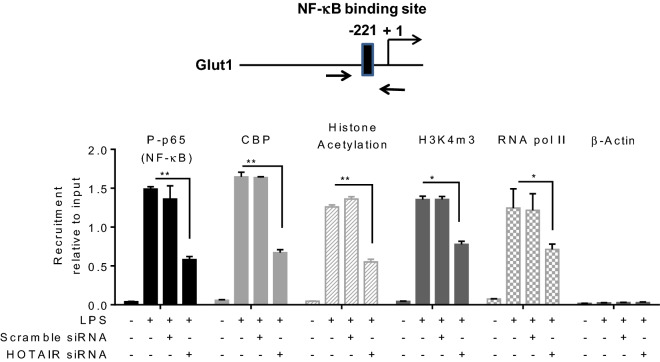
Knockdown of HOTAIR reduces the LPS-dependent recruitment of NF-κB on Glut1 promoter. RAW264.7 macrophage cells were transfected with HOTAIR and scramble-siRNA, then treated with LPS (1.5 h). Cells were then fixed with formaldehyde and subjected to ChIP assay using antibodies specific to phospho-p65, CBP, histone acetylation, H3K4m3, RNA pol II and β-actin (control). The immunoprecipitated DNA fragments were analyzed by qPCR using primers specific to the NF-κB binding regions on Glut1 promoter. The location of NF-κB binding site at the Glut1 promoter is shown in the top panel. Each experiment was repeated at least thrice (n = 3). Data represent mean ± SD; *p < 0.05, **p < 0.001.

The activation of transcription factors NF-κB plays key roles in the regulation of cytokines and pro-inflammatory genes during inflammation and immune response^4,7^. Upon inflammation response, the activated NF-κB translocate to the nucleus, binds to the target gene promoters, facilitate activators/coactivators recruitments and that allow chromatin modification and remodeling ultimately resulting in NF-κB target gene activation^76,77^. As HOTAIR regulates Glut1 expression and it also regulates NF-κB activation upon LPS-stimulation, we examined the LPS-induced enrichment of NF-κB to the Glut1 promoter and if this is regulated via HOTAIR. Glut1 promoter sequence analysis (as described by us previously^30,69^) revealed the presence of a putative NF-κB binding sites (GGGGATGTCT) upstream of the transcription start site (Fig. 4, top panel, this is in agreement with previous studies^78^) suggesting its potential regulation by NF-κB. Here, using chromatin immunoprecipitation (ChIP) assay, we have analyzed the NF-κB enrichment at the Glut1 promoter in an LPS-dependent manner and under HOTAIR-knockdown conditions^30,69^. Briefly, control, scramble-siRNA, and HOTAIR-siRNA-treated RAW264.7 cells were treated with LPS, fixed with formaldehyde, and then subjected to ChIP using antibodies against phosphorylated p65 (NF-κB subunit) and β-actin (control). ChIP DNA fragments were analyzed by qPCR using primers spanning the NF-κB binding site present in the Glut1 promoter. Interestingly, these analyses demonstrated that phospho-p65 (NF-κB) levels were enriched at the Glut1 promoter (NF-κB response element regions) upon treatment with LPS and this LPS-induced NF-κB binding was reduced upon HOTAIR-knockdown (treated with HOTAIR-siRNA followed by LPS) in the Glut1 promoter (Fig. 4, first panel). Scramble siRNA treatment has no significant impact on the LPS-induced enrichment of NF-κB at the Glut1 promoter. Neither LPS nor HOTAIR-knockdown has any significant impact on β-actin (antibody control) enrichment on the Glut1 promoter (Fig. 4, rightmost panel). Notably, along with NF-κB, there are other coactivators which may be associated with LPS-induced Glut1 expression. For example, histone acetyltransferase CBP (CREB binding protein) is known to interact with NF-κB to regulate NF-κB target genes^76,77,79^. Our ChIP analysis demonstrated that similar to NF-κB, the level of CBP was also enriched at Glut1 promoter and this LPS-induced CBP enrichment was alleviated upon knockdown of HOTAIR (Fig. 4). These observations suggest that HOTAIR plays key roles in LPS-induced NF-κB activation and hence the enrichment of NF-κB and its coactivators such CBP at the Glut1 promoter to regulate its expression in an LPS-dependent manner.

Along with activators and coactivators, histone H3K4-trimethylation and histone acetylation are also well known chromatin modifications associated with gene activation^80–82^. Our ChIP analysis demonstrated that levels of H3K4-trimethylation and histone acetylation as well as the level of RNA polymerase II (RNA pol II) were enriched at the Glut1 promoter in the presence of LPS and this was decreased upon HOTAIR knockdown (Fig. 4). This observation suggested that HOTAIR is required for promoter activation (H3K4-methylation and histone acetylation) of Glut1 and this is mediated via activation of NF-κB followed by recruitment of NF-κB and its coregulators including histone methyl-transferases and histone acetyl-transferases at the target gene promoters. The ChIP analysis demonstrated that LPS-induced expression of Glut1 is regulated via transcription factors NF-κB, CBP, and other coactivators and this is regulated by HOTAIR via regulation of NF-κB activation.

### HOTAIR regulates glucose uptake under LPS-simulation

**Figure 5:**
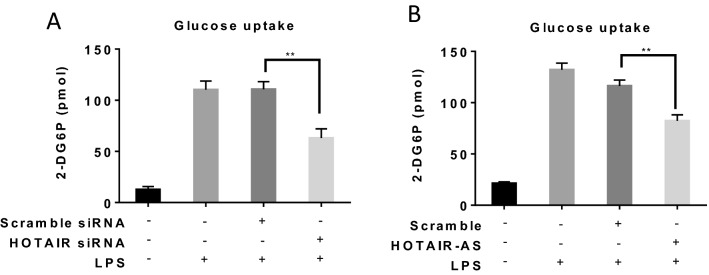
Knockdown of HOTAIR reduces the uptake of glucose in macrophages. RAW264.7 macrophages were treated with HOTAIR siRNA, scramble-siRNA, HOTAIR antisense or scramble-antisense oligonucleotides, separately, for 48 h and after overnight incubation, cells were stimulated with insulin (+/−), followed by 2-deoxyglucose addition for 20 min. 2-DG6P was oxidized and that generates NADPH, which was quantified calorimetrically (measured at 412 nm in a microplate reader). Panels A and B show the glucose uptake analysis under siRNA and antisense-meditated HOTAIR knockdown respectively. Each experiment was repeated at least thrice (n = 3). Data represent mean ± SD; *p < 0.05, **p < 0.001.

As HOTAIR regulated LPS-induced Glut1 expression in macrophage, we investigated if HOTAIR regulates the level of glucose uptake in macrophages during LPS stimulation and inflammation. Notably, Glut1 is a cytosolic protein and this translocates to the cell membrane and thus allows glucose uptake during glucose metabolism^83–85^. It is well-recognized that the level of glucose uptake and metabolism is increased during inflammation and this helps cells to tackle critical inflammatory situation^17,20^. Here, our studies demonstrate that Glut1 expression is increased upon LPS-stimulation in macrophage and this is regulated by HOTAIR. Therefore, we hypothesize that under LPS-stimulation condition, the level of glucose uptake will increase and this might be regulated via HOTAIR. To test this hypothesis, we measured the level of uptake of glucose (using a commercial kit) in macrophage cells under LPS treatment and in the presence and absence of HOTAIR-knockdowns. Briefly, RAW264.7 macrophages were treated with HOTAIR and scramble siRNAs for 48 h and then stimulated with insulin followed by 2-deoxyglucose (2-DG, a modified glucose) treatment. 2-DG is taken up by the cells by glucose transporter (Glut1 in our case) and gets phosphorylated to 2-DG6P. Once up taken by the cells, 2-DG cannot be further metabolized in glycolysis pathway and therefore will be accumulated inside the cells^19,45^. Accumulation of 2-DG6P is proportional to glucose uptake by the cells. The accumulated 2-DG6P level was determined by colorimetric reactions (as recommended by the kit)^19,45^. Interestingly, our results demonstrated that the level of glucose uptake is increased (10 folds) in macrophages upon treatment with LPS and the LPS-induced increased glucose uptake level is significantly reduced upon HOTAIR knockdown (HOTAIR-siRNA treatment) (Fig. 5A). Scramble-siRNA has no significant impacts on LPS-induced glucose uptake level. These observations demonstrate that HOTAIR regulates the LPS-induced glucose uptake during inflammation.

To further confirm the effects of HOTAIR on glucose uptake, we knocked down HOTAIR levels using HOTAIR-specific antisense oligonucleotide (a scramble antisense as used in parallel as a negative control) in RAW264.7 cells, then stimulated with insulin followed by 2-deoxyglucose (2-DG, a modified glucose) treatment and then measured the glucose uptake efficiency using similar experiments as described above (Fig. 5B), These results also demonstrated that antisense mediated knockdown of HOTAIR also downregulated the LPS-induced glucose uptake in macrophage (Fig. 5B). This observation in combination with the siRNA-mediated HOTAIR knockdown and glucose uptateke analysis results demonstrated that HOTAIR plays key roles in glucose uptake under inflammatory condition and this is likely mediated via induction of LPS-induced Glut1 expression.

### HOTAIR regulates upstream-regulators of glucose metabolism under LPS-simulation

**Figure 6:**
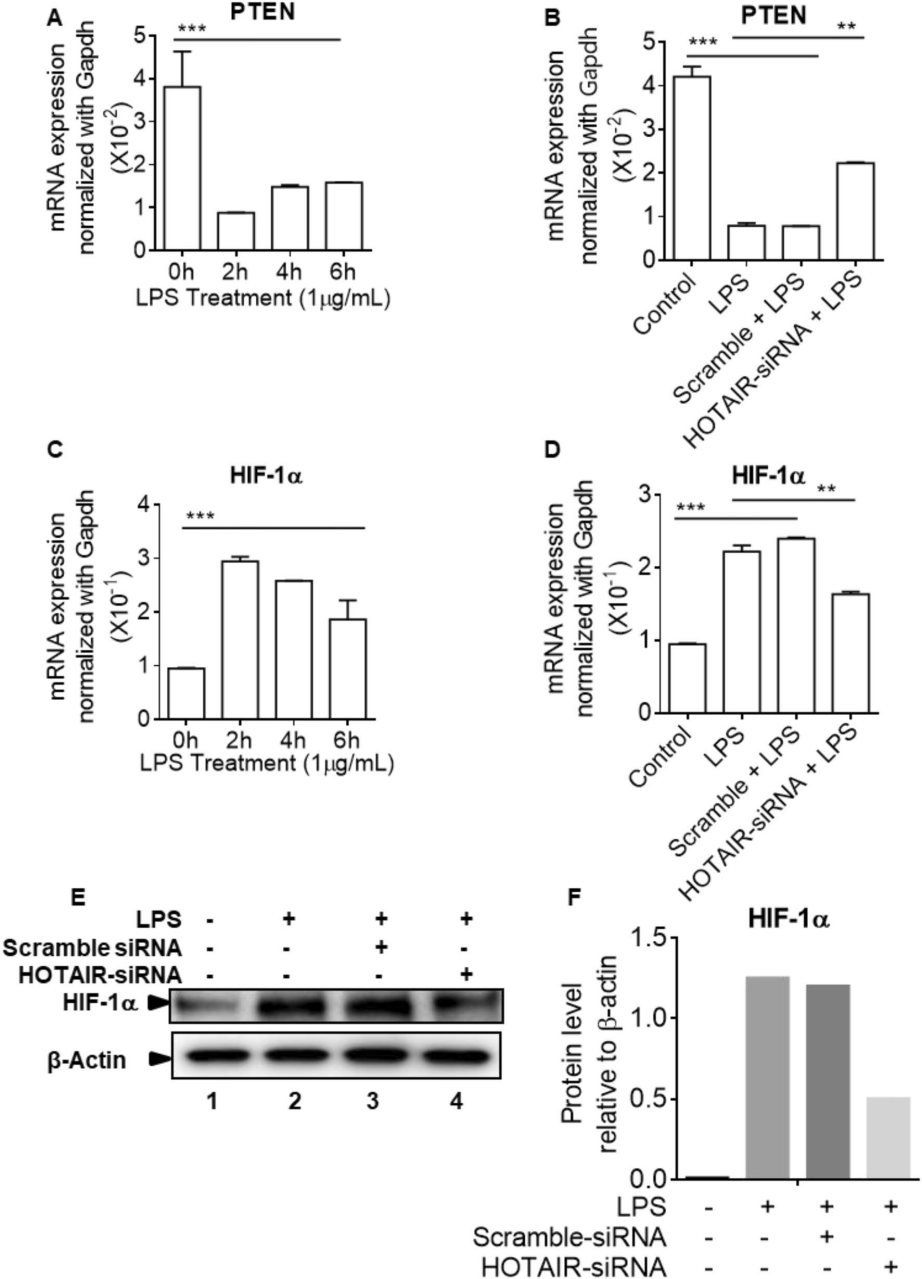
HOTAIR regulates upstream-regulators of glucose metabolism under LPS-simulation. (**A**,**C**): RAW264.7 cells were treated with LPS for varying times. RNA was analyzed by RT-qPCR for the expression of PTEN and HIF-1α. GAPDH was used as control. Data represent mean ± SD (n = 3); *p < 0.05, **p < 0.001, ***p < 0.0001. (**B**,**D**): RAW264.7 cells were transfected (48 h) with HOTAIR-siRNA and scramble siRNA followed by treatment with LPS. RNA was analyzed by RT-qPCR for the expression of PTEN (panel **B**) and HIF-1α (Panel **D**). GAPDH was used as control. Data represent mean ± SD (n = 3); *p < 0.05, **p < 0.001, ***p < 0.0001. (**E**,**F**) Proteins from HOTAIR-siRNA and scramble siRNA treatments (48 h) followed by 1 h LPS-treated RAW264.7 cells were analyzed by Western blotting using antibodies against HIF-1α and β-actin (loading control). The changes in amounts of HIF-1α have been quantified by ImageLab5.2.1 software and shown in panel **F**.

Glucose metabolism is a complex signaling process and there are many upstream signaling cascades that contribute to Glut expression, glucose uptake, and metabolism. Here we demonstrated that HOTAIR plays critical roles in the regulation of Glut1 expression and glucose uptake into macrophages under inflammation. We showed that HOTAIR activates NF-κB which contributes to Glut1 expression and glucose uptake. Notably, HOTAIR down regulates PTEN, which blocks the activation of PI3K/AKT signaling^86^. PTEN is a tumor suppressor which regulates cell growth and cell apoptosis^87^. Its often deleted, mutated and aberrantly expressed in a variety of cancer^88^. Studies have shown that HOTAIR inhibits PTEN gene expression by via methylation^89^. As PTEN is an upstream regulator of PI3K/AKT signaling which plays major roles in glucose metabolism, and PTEN, is regulated by HOTAIR in cancer cells, we examined if PTEN expression under LPS-stimulation in macrophage and if this is regulated by HOTAIR. RNA samples from the macrophages treated with LPS in the absence and presence of HOTAIR-knockdowns were analyzed by qPCR using PTEN primers. Interestingly, PTEN expression was down-regulated in macrophage upon LPS-stimulation and this was alleviated upon HOTAIR-knockdown (see Fig. 6A,B), suggesting HOTAIR mediated repression of PTEN gene expression.

Additionally, PTEN is known to block the activation of PI3K/AKT signaling and AKT activation stabilizes HIF-1α, which is involved in the induction of Glut1 expression. Glut1 is one of the HIF-1α target genes. PI3K/AKT signaling can also lead to activation of mTOR, which plays role in the glucose metabolism. As PTEN is repressed by HOTAIR under LPS-stimulation of macrophages, we also analyzed the HIF1α expression levels under LPS-stimulation and in the absence and presence of HOTAIR knockdowns. Here our analysis showed that, HIF-1α expression is elevated in macrophages upon LPS-treatment and this is decreased upon HOTAIR knockdown, both at the mRNA and proteins levels, suggesting critical roles of HOTAIR in HIF-1α expression during inflammatory response (Figs. 6C–F and S4). Thus, as HIF-1α is an upstream regulator of Glut1 and HOTAIR regulates HIF-1α, HOTAIR mediated regulation of Glut1 may follow diverse mechanism.

### HOTAIR regulates LPS-induced Glut1 expression and glucose uptake in primary bone marrow derived macrophages (BMDM)

**Figure 7:**
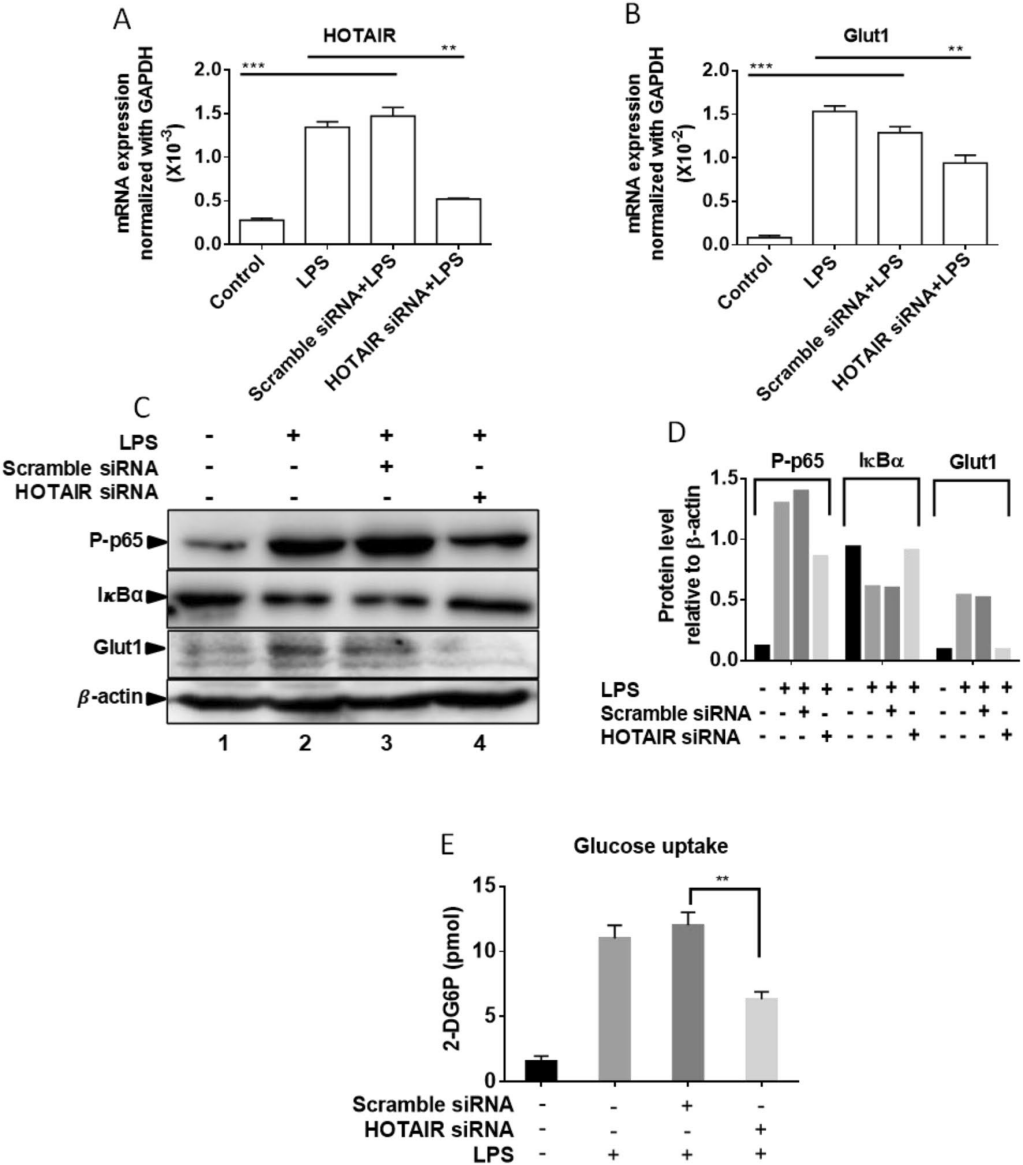
Glut1 expression is induced by LPS in primary macrophages (Bone marrow derived macrophages, BMDM). (**A**,**B**) BMDM cells were treated by HOTAIR siRNA and scramble siRNA followed by LPS treatment and RNA was extracted. The expression of HOTAIR and Glut1 was measured by real time PCR. Data represent mean ± SD (n = 3); *p < 0.05, **p < 0.001, ***p < 0.0001. (**C**,**D**) BMDM cells were treated by HOTAIR siRNA and scramble siRNA and treated with LPS. Proteins were analyzed by Western blotting using antibodies against phospho-p65 (NF-κB subunit), IκBα, Glut1, and β-actin (loading control). The changes in amounts of NF-κB, IκBα, and Glut1 have been quantified by ImageLab5.2.1 software is shown in panel (**D**). (**E**) BMDM cells were treated with HOTAIR and scramble-siRNA for 48 h and after overnight incubation, cells were stimulated with insulin (+/−), followed by 2-deoxyglucose addition for 20 min. 2-DG6P was oxidized and that generates NADPH, which was quantified calorimetrically (measured at 412 nm in a microplate reader). Each experiment was repeated at least thrice (n = 3). Data represent mean ± SD; *p < 0.05, **p < 0.001.

We investigated further the importance of HOTAIR in Glut1 regulation and glucose uptakes in primary macrophages (bone marrow derived macrophage, BMDM). Briefly, BMDM cells were isolated from mouse bone marrow and maintained as described by us previously^30,65,71^. BMDMs were treated with LPS (1 µg/mL, for 4 h) in the presence and absence of HOTAIR-siRNA and scramble-siRNA transfections. RNA was analyzed by RT-qPCR. Interestingly, the level of Glut1 as well as HOTAIR expression was induced upon treatment with LPS in BMDMs (Fig. 7A,B). Application of HOTAIR siRNA resulted in significant knockdown of LPS-induced HOTAIR expression level (Fig. 7A) and this also resulted in a decrease in LPS-induced Glut1 expression (Fig. 7B). Scramble siRNA has no significant impacts on LPS-induced HOTAIR or Glut1 expression (Fig. 7A,B). These observations demonstrated that Glut1 expression is induced upon LPS-stimulation in primary macrophages and this is regulated via lncRNA HOTAIR.

HOTAIR-knocked down and LPS-treated BMDM cells were also analyzed by Western blot to understand the function of HOTAIR for Glut1 expression and NF-κB activation. Interestingly, we found that IκBα protein levels were higher in the control BMDM (no LPS) and this was decreased upon treatment with LPS (compare lanes 1 and 2, Figs. 7C and S5). The level of NF-κB (phospo-p65) protein was increased upon treatment with LPS (compare lanes 1 and 2, Figs. 7C and S5). Interestingly, upon HOTAIR-knockdown, the level of LPS-induced decrease in IκBα protein level was rescued significantly while LPS-induced phospho-p65 NF-κB protein level was decreased (compare lanes 1, 2 and 4, Figs. 6C and S5). Scramble-siRNA has no significant impacts on LPS-induced expression of IκBα and NF-κB (phospho-p65) protein levels. Importantly, Glut1 protein level is also induced by LPS and this was reduced upon treatment with HOTAIR-knockdown (HOTAIR-siRNA treatment, Fig. 7C). Quantification of Western blot showing the expression of IκBα, NF-κB (phospho-p65) and Glut1 protein levels are shown in Fig. 7D.

We also performed the glucose uptake assay (using a commercial kit) in BMDMs under LPS treatment and in the presence or absence of HOTAIR-knockdowns, using similar procedure as described in Fig. 5. Briefly, BMDMs were treated with HOTAIR and scramble siRNAs (48 h), then stimulated with insulin followed by treatment with 2-deoxyglucose (2-DG) which (once uptaken) gets phosphorylated to 2-DG6P and accumulated inside cells^19,45^. The accumulated 2-DG6P level was determined by colorimetric reactions (as recommended by the kit)^19,45^. Our results demonstrated that the level of glucose uptake is increased in BMDM upon treatment with LPS and the LPS-induced increased glucose uptake level is significantly reduced upon HOTAIR knockdown (HOTAIR-siRNA treatment) (Fig. 7E). Scramble siRNA has no significant impacts on LPS-induced glucose uptake level. These observations based on primary macrophage analysis further demonstrate that HOTAIR plays a critical role in NF-κB activation, Glut1expression, and glucose uptake during inflammation and immune response in macrophages.

## Discussion

Inflammation is the intrinsic immune response of the body towards invading pathogens^90–92^. The inflammatory response causes activation of immune cells such as leukocytes (aka white blood cells), T cells, and B cells, and induces production of inflammatory mediators such as cytokines and chemokines to fight against injury or infection^21,93,94^. However, uncontrolled and continued inflammation drives the development of many human diseases, including metabolic diseases, obesity, diabetes, autoimmune disorders, neurological disorder, and cancer^16,42^. The signaling process associated with inflammation and immune response is very complex. Our understanding of inflammation and immune response are largely limited to genomic markers and proteins-based factors that are involved in transcription, phosphorylation, ubiquitination, and protein–protein interactions^93,95,96^. Even with the huge amount research and options of available therapeutics, many inflammatory diseases still cannot be treated effectively^2,80,97^. Therefore, understanding the detailed signaling mechanism associated inflammation and immune response is critical for developing effective therapies. Emerging evidences suggest that noncoding RNAs (ncRNAs), play critical roles in various cellular and physiological process including in gene expression, cell differentiation, development, and their dysregulation contributes to critical human disease^98–100^. NcRNAs are recently discovered highly heterogeneous group of transcripts that are coded by the genome, transcribed, but mostly remain untranslated^33,35,52^. Previously, ncRNAs were considered to be a consequence of transcriptional noise. Recent studies, however, suggest that ncRNAs have distinct cellular functions and are involved in different biological processes^101–103^. In a recent study, we discovered that long noncoding RNA HOTAIR plays critical roles in NF-κB activation, cytokine regulation, inflammation, and immune response^30^.

Activation of immune cells in response to infection or other stressors is a metabolically expensive event^16,31,61,104^. The immune cells preferentially meet their energy needs by metabolizing glucose^61,62,105^. Furthermore, glucose metabolism is involved in mounting inflammatory immune responses^18,63,106^. Interestingly, the inflammatory response induces expression of glucose metabolizing enzymes and related factors such as Glut1, glucose-6-phosphate dehydrogenase, hexokinase, and increase glycolysis^107,108^. Therefore, we investigated if HOTAIR is involved in inflammation induced metabolic reprogramming. Our studies demonstrated that, along with proinflammatory genes such as IL-6 and iNOS, the inflammatory lncRNA HOTAIR and Glut1 expression are induced in macrophages upon LPS-stimulation and Glut1 is the major glucose transporter in expressed macrophage and potentially associated with glucose uptake and metabolism during inflammation.

NF-κB activation plays a central role in immune response and inflammation^27,28,109^. NF-κB activation induces expression of NF-κB regulated cytokines and pro-inflammatory genes^90,93^. Though it is well known that activation of immune cells in response to infection or other stressors is a metabolically expensive event and metabolizing glucose is a major source of energy to meet the energy requirement of inflammatory response, the detailed signaling mechanism by which NF-κB activation is integrated to glucose metabolism remains elusive. Our mechanistic studies demonstrated that the inhibition of NF-κB (via inhibition of IKKβ kinase by SC-514) significantly reduces LSP-induced Glut1 expression, suggesting potential roles of NF-κB in Glut1 expression during inflammation.

Our recent studies also demonstrated that lncRNA HOTAIR regulates NF-κB activation via degradation of IκBα^30^. As LPS-induced Glut1 expression is regulated via NF-κB activation which is critically regulated via HOTAIR, we explored the potential role of HOTAIR in LPS-induced Glut1 expression. Indeed our studies demonstrated that siRNA-mediated knockdown of HOTAIR suppressed the LPS-induced expression of Glut1 in macrophage. This is likely because of reduced NF-κB activation under HOTAIR knockdown condition. Importantly, NF-κB activation regulates cytokines and pro-inflammatory genes expression via binding to its target gene promoters, followed by recruitment of activators, coactivators, chromatin modification and remodeling^110–112^. Typical NF-κB regulated genes (such as cytokines) promoters contain binding sites for transcription factor NF-κB and upon inflammation, activated NF-κB gets recruited to the target gene promoters which aids the recruitment of activators, coactivators and chromatin modifiers, resulting in their gene activation^76,110,113^. Our analysis demonstrated that similar to well-known NF-κB target genes (such as, IL-6, iNOS, etc.), the Glut1 promoter has NF-κB binding site close to the transcription start site. Our ChIP analysis indeed demonstrated that upon LPS-stimulation, NF-κB is activated and is enriched at the Glut1 promoter (NF-κB response element). Along with NF-κB, histone acetyl-transferase and NF-κB associated activator, CBP, is enriched at the Glut1 promoter in response to LPS-treatment^76,77^. Additionally, gene activation associated chromatin modifications such as histone H3K4-trimethylation and histone acetylation levels are also increased at the Glut1 promoter in an LPS-dependent manner. Importantly, these LPS-induced enrichments in NF-κB, CBP, H3K4-trimethyl, histone acetylation, and RNA polymerase II levels at the Glut1 promoter, were reduced upon knockdown of HOTAIR. These observations further demonstrate critical roles of HOTAIR in LPS-induced Glut1 expression. HOTAIR promotes IκBα degradation which makes NF-κB free from its inhibitory effect and induces availability of activated NF-κB for binding to its target genes promoter such as Glut1 promoter. Thus, our observations suggest further about the crucial roles of HOTAIR in LPS-induced activation of NF-κB and Glut1 expression under inflammation.

As HOTAIR controls the expression glucose transporter Glut1 under LPS-stimulation and Glut1 being the major glucose transporter in macrophage, we explored if HOTAIR also controls the glucose uptake in macrophages during inflammatory responses. Our studies demonstrate that glucose uptake efficiency is increased in macrophages upon LPS stimulation and this is suppressed under HOTAIR-knockdown condition. These observations suggest that HOTAIR is a critical player in Glut1 expression and glucose uptake and potentially regulate glucose metabolism during inflammation. Importantly, glucose metabolism and signaling is complex and involves many upstream regulators and signaling cascade. For example, PI3K/AKT signaling activation is critical to glucose metabolism and PI3K/AKT activation is blocked by PTEN^86^, which acts as a tumor suppressor to regulate cell growth and cell apoptosis^87^. In this study, we demonstrated that HOTAIR suppresses PTEN gene expression during LPS-induced inflammation in macrophage. By performing RNA pulldown assay, Zhang et al., have demonstrated that HOTAIR negatively regulate PTEN via directly binding with it^114^. They also showed that HOTAIR activates the PI3K/Akt pathway to promote Endometrium Cancer progression by suppressing PTEN in vivo^114^. Furthermore, AKT activation has been reported to stabilize HIF-1α, which is involved in the induction of Glut1 expression^115^. Indeed, Glut1 is one of the HIF-1α target genes^116^. In this study we showed that HOTAIR induces HIF-1α expression. Our observation also suggests that HIF-1α is involved in Glut1 expression. PI3K/AKT signaling can also lead to activation of mTOR, which also plays role in the glucose metabolism. Our studies demonstrated that HOTAIR is also involved in the down regulation of PTEN and increase of HIF-1α.

**Figure 8:**
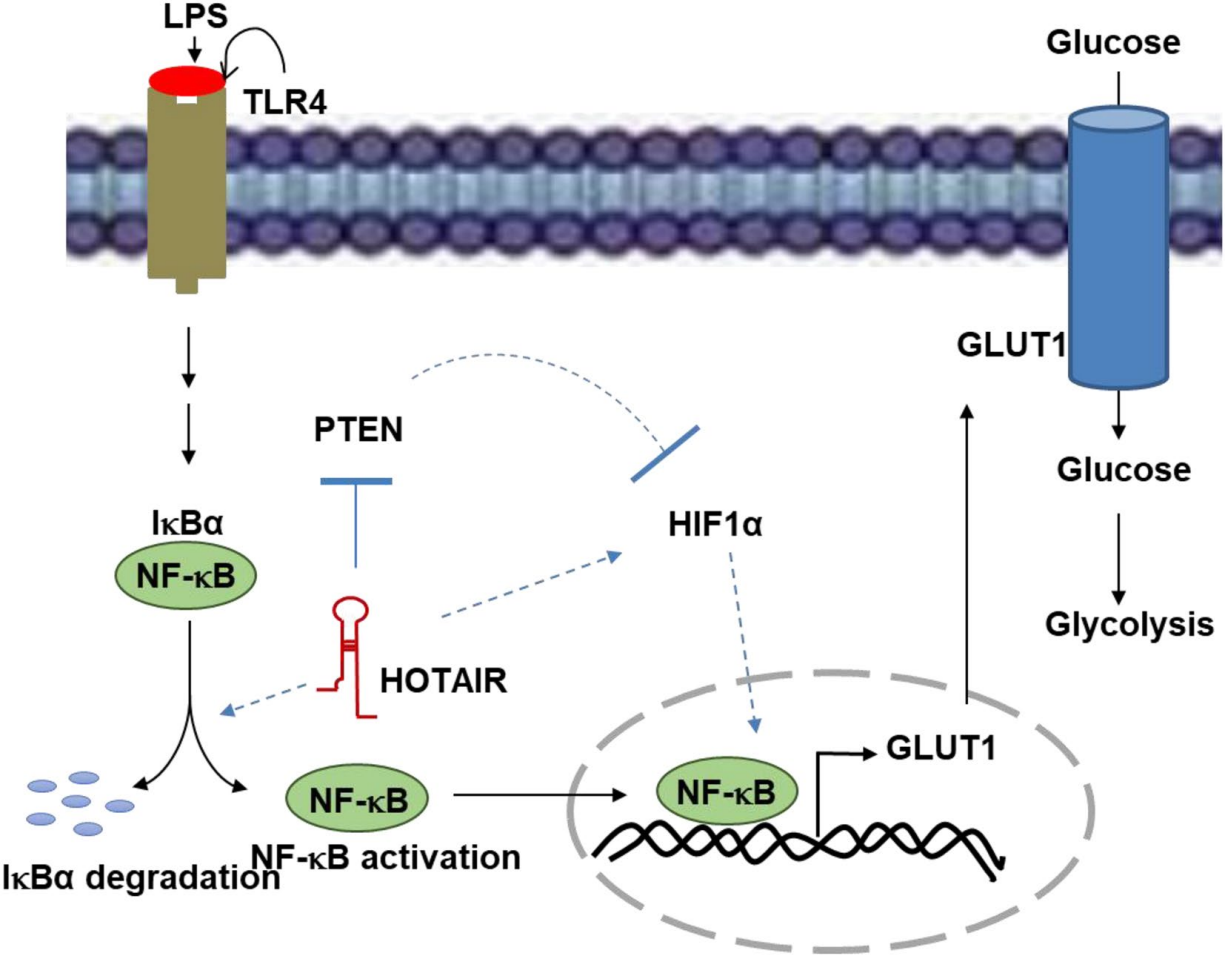
Proposed model for HOTAIR mediated regulation of glucose metabolism during inflammation. When TLR4 senses LPS, NF-κB is activated that induces HOTAIR expression. In turn, HOTAIR facilitates IκBα degradation and enhances NF-κB activation, its nuclear translocation, and binding to NF-κB regulated Glut1 gene promoter inducing its expressions. Overexpressed Glut1 is translocated to membrane, allows increased glucose uptake and glucose metabolism, during inflammation and immune response. PTEN is repressed by HOTAIR under LPS-stimulation. Thereby, HOTAIR relieved PTEN mediated suppression of HIF-1α. Thus HOTAIR mediated repression of PTEN and induction of HIF-1α under LPS-stimulation, contribute increased Glut1 expression and glucose metabolism.

Beyond the studies in cultured macrophages (RAW264.7), we extended our studies to primary cells, BMDMs. Studies in BMDMs also further supported our observations that HOTAIR plays critical roles in NF-κB activation, glucose transporter (Glut1) expression and glucose update during LPS-stimulation, suggesting it potential roles in metabolic reprogramming during inflammation and immune response. Notably, a recent study reported that HOTAIR promotes glycolysis in hepatocytes and this further supports our observation of about the potential roles of HOTAIR in regulation of glucose metabolism^117^. A model, showing the potential roles of HOTAIR in regulation of glucose uptake via inducing Glut1 expression through IκBα degradation and NF-κB activation, its translocation into the nucleus, and binding to the Glut1 promoter is shown in Fig. 8. Importantly, glucose metabolism and inflammation in macrophages are closely associated with variety chronic metabolic disorder such as obesity and diabetes^118,119^. The signaling mechanism linking inflammation with metabolic disorders are complex and identifying novel regulatory mechanism for the inflammation associated signaling is critical towards understanding the pathogenesis of metabolic disorders and also for developing novel therapeutics. Notably, independent studies from other laboratories also demonstrated that Glut1 expression is controlled by NF-κB activation. For example, in liver cancer, Glut1 expression is induced by increased NF-κB which is mediated via oncogenic factor LAMTOR5^78^. Another reported that transcription factor Sp1 coordinates with NF-κB and regulates Glut1 expression in response to reactive oxygen specific via activation of MAPK dependent pathways^120,121^. Therefore, our studies showing the critical roles of lncRNA HOTAIR in NF-κB activation Glut1 expression and glucose uptake under LPS-induced inflammation in macrophages reveals a novel signaling pathway by which is lncRNAs may influence glucose metabolism and may induce metabolic reprogramming in macrophages during imflammation and immune response and therefore, this may serve as a novel therapeutic avenue in the treatment of inflammatory, immune, and metabolic disease. Our studies further suggest that long noncoding RNA are integral components of immune signaling and inflammation and there are novel therapeutic targets for immune and inflammatory diseases.

## Supplementary Information

Supplementary Figures.
