# Supplementary material for: LncRNA HOTAIR regulates glucose transporter Glut1 expression and glucose uptake in macrophages during inflammation
Source: Sci Rep. 2021 Jan 8;11:232. doi: 10.1038/s41598-020-80291-4 (PMC7794310; doi:10.1038/s41598-020-80291-4)
Supplement: Supplementary file 1 — Supplementary Figures. [file 41598_2020_80291_MOESM1_ESM.pdf]

Supplementary information

LncRNA HOTAIR regulates glucose transporter Glut1 expression and glucose uptake in macrophages during inflammation

Monira Obaid[1], S. M. Nashir Udden[2], Prasanna Alluri[2], and Subhrangsu S. Mandal[1]*

[1]Department of Chemistry and Biochemistry, The University of Texas at Arlington, Arlington, Texas 76019. [2]Department of Radiation oncology, The University of Texas Southwestern Medical Center, Dallas, Texas 75390.

*Corresponding author. E-mail: smandal@uta.edu; Fax: 817-272-3808

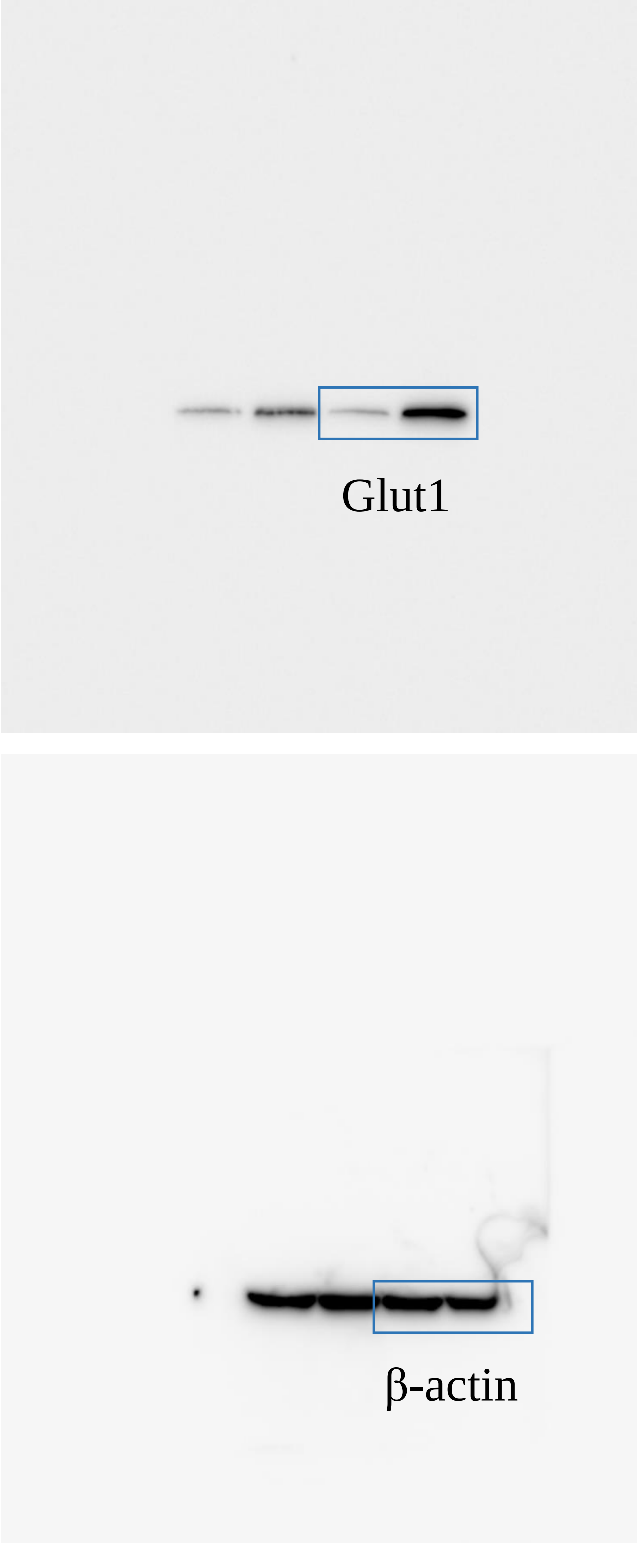

Figure S1. LPS induces Glut1 expression in macrophages. RAW264.7 were treated with LPS (1 μg/ml) and kept untreated. Protein was collected and the protein was resolved on SDS-PAGE and immunoblotted with antibody against the indicated proteins. Figure 1F was cropped from the above images.

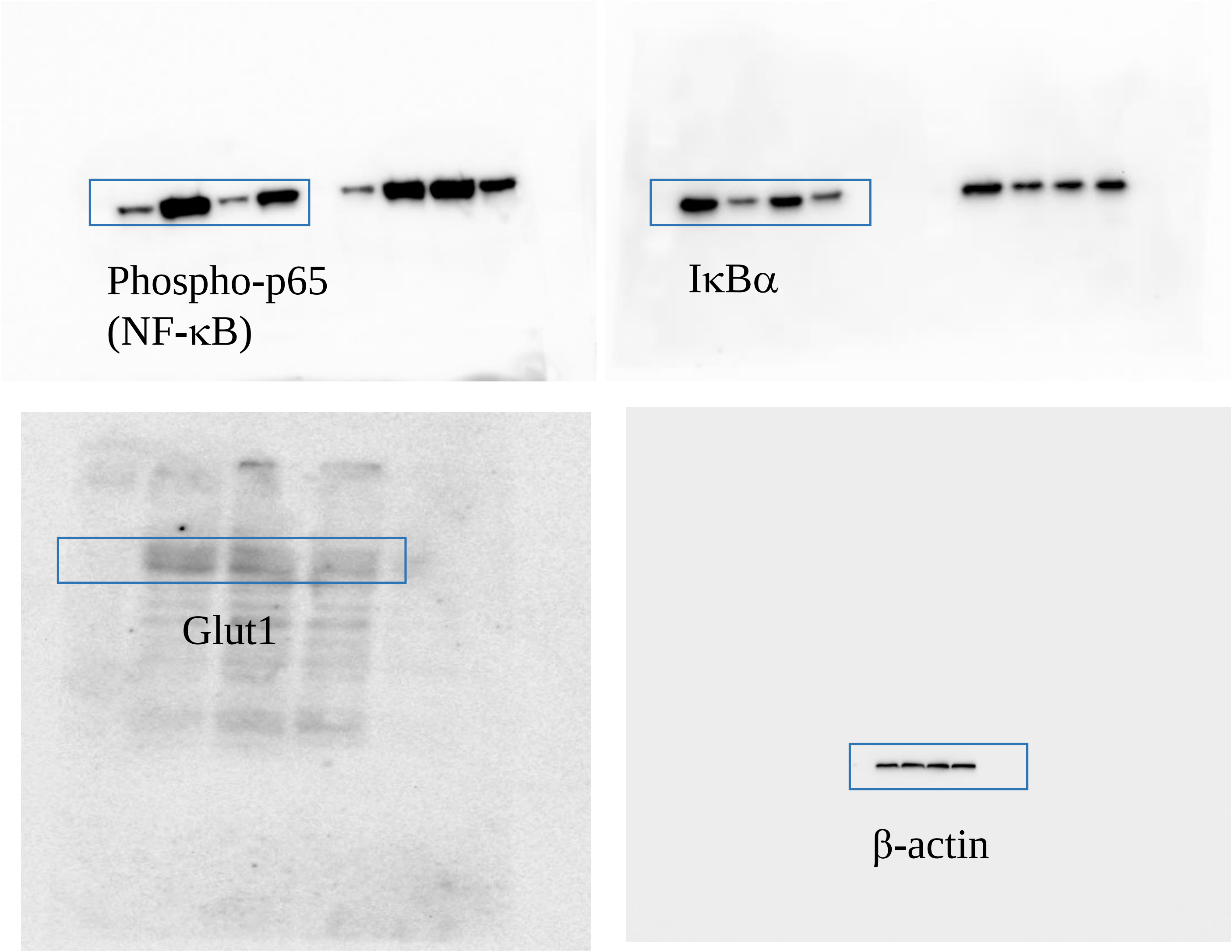

Figure S2. Inhibition of NF-κB by IKK*β* inhibitor (SC-514). RAW264.7 were pre-treated with IKKβ inhibitor (25μM, SC-514, Sigma) for 1h, and after then treated with LPS (1 μg/ml), IKKβ inhibitor individually and combinedly, and kept untreated for 1h. Protein was collected and the protein was resolved on SDS-PAGE and immunoblotted with antibody against the indicated proteins. Figure 2A was cropped from the above images.

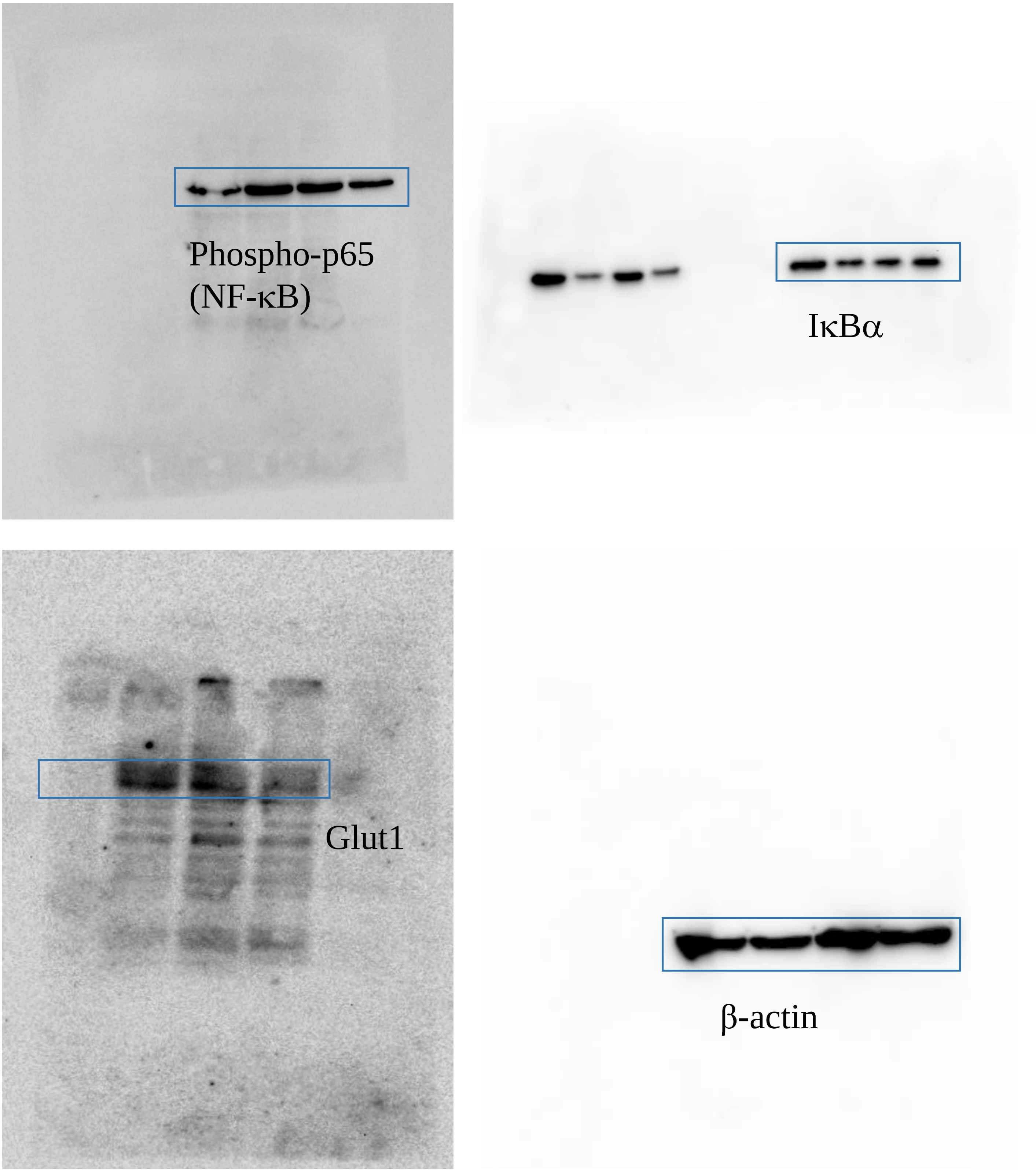

Figure S3. SiRNA mediated knockdown of HOTAIR. HOTAIR was silenced in RAW264.7 macrophages by using HOTAIR specific SiRNA for 48h following transfection, cells were stimulated with LPS for 1h or kept untreated, harvested and protein was isolated. The protein was resolved on SDS-PAGE and immunoblotted with antibody against the indicated proteins. Figure 3C was cropped from the above images.

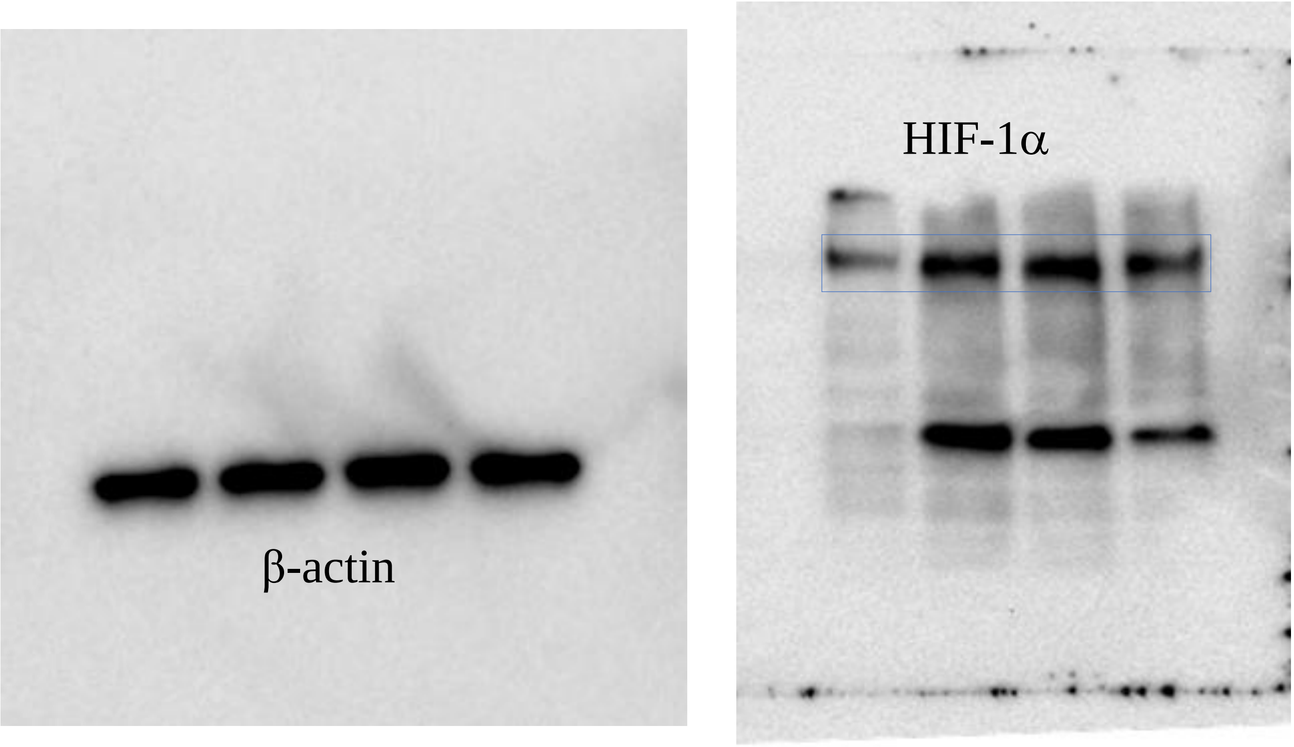

Figure S4. SiRNA mediated knockdown of HOTAIR. HOTAIR was silenced in RAW264.7 macrophages by using HOTAIR specific SiRNA for 48h following transfection, cells were stimulated with LPS for 1h or kept untreated, harvested and protein was isolated. The protein was resolved on SDS-PAGE and immunoblotted with antibody against the indicated proteins. Figure 6E was cropped from the above images.

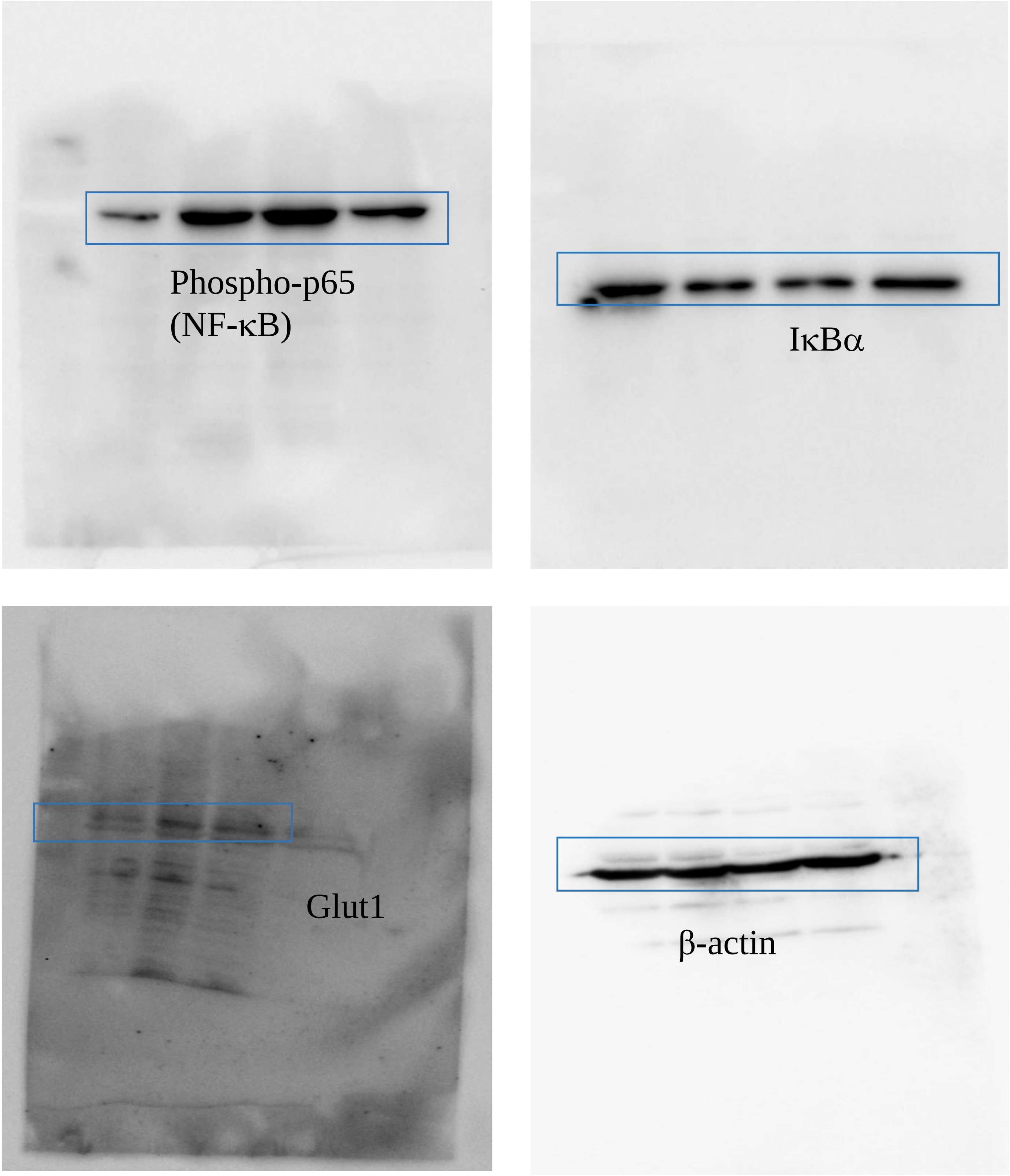

Figure S5. SiRNA mediated knockdown of HOTAIR. HOTAIR was silenced in bone marrow derived macrophages (BMDM) by using HOTAIR specific SiRNA for 48h following transfection, cells were stimulated with LPS for 1h or kept untreated, harvested and protein was isolated. The protein was resolved on SDS-PAGE and immunoblotted with antibody against the indicated proteins. Figure 7C was cropped from the above images.
